# Characterizing indigenous plant growth promoting bacteria and their synergistic effects with organic and chemical fertilizers on wheat (*Triticum aestivum*)

**DOI:** 10.3389/fpls.2023.1232271

**Published:** 2023-08-16

**Authors:** Israr Asghar, Maqsood Ahmed, Muhammad Ansar Farooq, Muhammad Ishtiaq, Muhammad Arshad, Muhammad Akram, Adnan Umair, Abdulwahed Fahad Alrefaei, Muhammad Yousuf Jat Baloch, Aamna Naeem

**Affiliations:** ^1^ Department of Biotechnology, Mirpur University of Science and Technology (MUST), Mirpur, Pakistan; ^2^ Institute of Environmental Sciences and Engineering, School of Civil and Environmental Engineering, National University of Sciences and Technology (NUST), Islamabad, Pakistan; ^3^ Department of Botany, Mirpur University of Science and Technology (MUST), Bhimber, Pakistan; ^4^ Department of Environmental Sciences, COMSATS University Islamabad (CUI), Vehari, Pakistan; ^5^ Department of Agriculture, Research wing, Soil and Water Testing Laboratory, Sialkot, Pakistan; ^6^ Department of Zoology, College of Science, King Saud University, Riyadh, Saudi Arabia; ^7^ College of New Energy and Environment, Jilin University, Changchun, China

**Keywords:** plant growth promoting bacteria, chemical fertilizers, organic fertilizers, wheat, integrated nutrient management

## Abstract

The excessive use of chemical fertilizers is deteriorating both the environment and soil, making it a big challenge faced by sustainable agriculture. To assist the efforts for the solution of this burning issue, nine different potential native strains of plant growth-promoting bacteria (PGPB) namely, SA-1(*Bacillus subtilis)*, SA-5 (*Stenotrophomonas humi)*,SA-7(*Azospirillum brasilense*), BH-1(*Azospirillum oryzae*), BH-7(*Azotobacter armeniacus*), BH-8(*Rhizobium pusense*), BA-3(*Azospirillum zeae*), BA-6(*Rhizobium pusense*), and BA-7(*Pseudomonas fragi*) were isolated that were characterized morphologically, biochemically and molecularly on the basis of 16S rRNA sequencing. Furthermore, the capability of indigenous PGPB in wheat (*Triticum aestivum*, Chakwal-50) under control, DAP+FYM, SA_-1,5,7_, BH_-1,7,8_, BA_-3,6,7_, DAP+ FYM + SA_-1,5,7_, DAP+FYM+ BH_-1,7,8_ and DAP+FYM+ BA_-3,6,7_ treatments was assessed in a randomized complete block design (RCBD). The results of the study showed that there was a significant increase in plant growth, nutrients, quality parameters, crop yield, and soil nutrients at three depths under SA_-1,5,7_, BH_-1,7,8_, and BA_-3,6,7_ in combination with DAP+FYM. Out of all these treatments, DAP+ FYM + BA_-3,6,7_ was found to be the most efficient for wheat growth having the highest 1000-grain weight of 55.1 g. The highest values for plant height, no. of grains/spike, spike length, shoot length, root length, shoot dry weight, root dry weight, 1000 grain weight, biological yield, and economic yield were found to be 90.7 cm, 87.7 cm, 7.20 cm, 53.5 cm, 33.5 cm, 4.87 g, 1.32 g, 55.1 g, 8209 kg/h, and 4572 kg/h, respectively, in the DAP+FYM+BA treatment. The DAP+FYM+BA treatment had the highest values of TN (1.68 µg/mL), P (0.38%), and K (1.33%). Likewise, the value of mean protein (10.5%), carbohydrate (75%), lipid (2.5%), and available P (4.68 ppm) was also highest in the DAP+FYM+BA combination. C:P was found to be significantly highest (20.7) in BA alone but was significantly lowest (11.9) in DAP+FYM+BA. Hence, the integration of strains BA-3, BA-5, and BA-7 in fertilizers can be regarded as the most suitable choice for agricultural growth in the sub-mountainous lower region of AJK. This could serve as the best choice for sustainable wheat growth and improved soil fertility with lesser impacts on the environment.

## 1 Introduction

The world population has been estimated to reach 9.1 billion by the year 2050, as per the projections of the Food and Agriculture Organization (FAO) in 2009 (FAO, 2009). Wheat, being a major grain crop, would be thus required in large quantities, with estimates suggesting a surge of up to 70% in wheat demand (Godfray et al., 2010). This increased demand can only be fulfilled by significant improvements in crop production. Over the past five decades, the excessive application of fertilizers to boost crop yields has completely ignored the impacts of such activities on the environment (Adesemoye et al., 2009; Souza et al., 2015; Jat Baloch et al., 2023). There is thus an urgent need to utilize innovative techniques that increase the productivity of crops and at the same time mitigate the adverse impacts on the environment such as ecological destruction and the release of hazardous gases (F.A.O, 2009).

Sustainable crop production is particularly important in the case of wheat production as this cereal crop is a primary dietary staple around the world, followed by rice and corn fulfilling the remaining dietary energy requirements of humans (Food and Agriculture Organization, 2014). In some areas of the world such as the Mediterranean, a special variety of wheat (Triticum *turgidum*) is consumed as an essential part of the diet on a daily basis (Troccoli et al., 2000). In Pakistan, wheat is a staple food crop that is produced in majority. A wheat-maize cropping pattern has been adopted here under the usage of chemical fertilizers for the last 50 years (Majeed et al., 2015; Jat Baloch et al., 2021; Jat Baloch et al., 2022). However, it is impossible to achieve the long-lasting targets of highly improved crop production by only relying on the use of chemical fertilizers without any combination (Sood et al., 2018; Khan et al., 2022).

The consistent use of chemical fertilizers is not only a grave environmental concern in the form of soil, water, and air pollution but is also a financial concern for farmers due to the ever-increasing price of fertilizers (Majeed et al., 2015). The practical application of potential biotechnological approaches to minimize the degrading effects of chemical fertilizers on the environment has become the main focus of a group of agricultural communities that mainly includes researchers and farmers. Integrated crop management is the best approach to achieve sustainable agriculture which is possible through green biotechnologies that are considered new and modern strategies. These novel management practices reduce the excessive use of chemical fertilizers, improve the ability of crops to uptake nutrients from the soil, and monitor biological complex mechanisms (Hirel et al., 2011; Singh and Ryan, 2015).

The consistent application of chemical fertilizers to crops without any limit can be minimized by adopting more efficient and progressive approaches which are known as microbial biotechnologies and are more acceptable for environmental protection with great capability for plant improvement in the right manner (Kumari et al., 2019; Niedbała et al., 2020). Integrated nutrient management (INM) may be a well-known crop managerial technique to promote wheat in the sense of both crop production and quality as well as to minimize the application of chemical fertilizers which ultimately controls pollution and degradation of the environment, thereby resulting in more persistent crop yield (Sedri et al., 2022).

The integration of bio-fertilizers such as plant growth-promoting bacteria (PGPB) in wheat positively influences the basic biochemical properties of soil such as a considerable increase in the concentration of major soil nutrients, soil microbial biomass, and soil microbial and enzymatic activities. PGPB belong to the free-living group of soil bacteria which improve both plant growth and crop yield when they are applied in different ways (Kumar et al., 2014). During both pot and field experiments, various PGPB strains such as *Pseudomonas fluorescens*, *B. megaterium*, *A. chlorophenolicus*, and *Enterobacter* have been found to enhance the growth and yield of wheat by significantly improving root weight, number of tillers per plant, grain yield, and straw yield (Shaharoona et al., 2008; Kumar et al., 2014). They also produce some important biochemicals such as siderophores, antibiotics, enzymes, and fungicides which are useful against different pathogens and compete with harmful microorganisms (Dey et al., 2004; Lucy et al., 2004). The native strains of PGPB are the best choice for integration as bio-fertilizer to enhance crop productivity without obstructing soil fertility because indigenous PGPB have great potential to retain soil nutritional status and can easily adapt to the local environment (Sood et al., 2018). The integrated application in all three forms of bio, organic, and chemical fertilizers such as PGPB, decomposed manure, and synthetic mineral fertilizers is most effective at improving overall crop production by considerably enhancing different parameters (Mihoub et al., 2022).

The manufacture of more valuable bio-fertilizer products from the mixture of biochemically decomposed material and PGPB which are living in the soil independently and are engaged to boost up the rooting system in an active or passive way (Mayak et al., 1999), ultimately leads towards plant enhancement (Glick et al., 1995; Mayak et al., 1999). PGPB with multiple features are also viable to promote plant development under unfavorable ecology with great limitations (Oleńska et al., 2020). They facilitate converting unavailable N and P into available forms through biological processes and the production of plant growth hormones and a variety of nutrient particles. The microbial technology, i.e., PGPB, facilitates the more prominent tool of integrated management systems that is responsible for the persistence of the crop yield procedures (Ahemad and Kibret, 2014). PGPB belong to a vast variety of genera such as *Azospirillum*, *Azotobacter*, *Bacillus*, *Pseudomonas*, and *Rhizobium* (Di Benedetto et al., 2017) which are present near the rooting system either independently or attached closely to the roots (Basu et al., 2021). The various PGPB strains, such as B2, BIS2, and SIR1, isolated from soil when applied with different doses of N and P fertilizers give maximum benefits and nutrients for the growth of wheat. One of the most beneficial strains for wheat yield has been found to be B2 (*Serratia* species) when co-applied with 80% Nitrogen Phosphorous fertilizer (Sood et al., 2018). Likewise, PGPB strains Pa (*Pantoea agglomerans)* and B25 (*Bacillus thuringiensis*) isolated from wheat fields have been found to have highly positive effects on wheat seed priming and their growth under stressed as well as non-stressed environments (Saadaoui et al., 2022). Hence, the various PGPB strains play a crucial role in promoting the effective growth of wheat under favorable as well as non-favorable soil conditions and help overcome the issues initiated due to the usage of hazardous chemical fertilizers.

The above discussion helped us to understand that excessive application of chemical fertilizers alone poses not only a deleterious effect on the environment but is also an over burden on poor local farmers due to heavy costs. Chemical fertilizers also degrade soil properties and disturb the balance of soil nutrients. The utilization of indigenous PGPB strains that are derived from the soil, rhizosphere, or endosphere of wheat crops in a proper way is the most sustainable, cost-effective, and suitable solution for local farmers. The beneficial effects of these PGPB strains for enhanced wheat productivity are evident when they are used in combination with other organic or inorganic fertilizers. Hence, it is necessary that these PGPB strains be isolated, identified, and utilized as bio-fertilizers for wheat as well as other crops. Unfortunately, the farmers of the region under study are unaware of this advanced novel approach in biotechnology. Keeping in view the above-mentioned importance of PGPB strains and their potential to overcome crop productivity issues in a given region, a study was conducted to (i) characterize and use indigenous PGPB strains for the improvement of sustainable agriculture; (ii) assess the effect of PGPB interaction with different doses of organic and chemical fertilizers on the yield, growth, and quality of wheat as well as on soil fertility.

## 2 Materials and methods

### 2.1 Experimental site

A field experiment was conducted to access the performance of indigenous PGPB in wheat, at Tehsil Samahni of District Bhimber AJK - previously under long-term wheat (*Triticum aestivum*) and maize (*Zea may*s) cultivation (Hussain, 2009). The northern side of Azad Jammu and Kashmir (AJK) is connected with the lower region of the Hindu Kush Himalaya (HKH). This part of AJK consists of large ranges of giant mountains while the southern part is comparatively less mountainous with plain areas. The southern region is fertile, making it suitable for the growth of every type of vegetation including fruits, vegetables, legumes, and cereal crops. Whereas the northern region possesses very small and narrow stretches of plain lands where few crops with great limitations are cultivated but is richest in fruit and forestry produce (Wikipedia, 2019).

Tehsil Samahni is in a part of the southern region with a combination of high mountains and low valleys that can produce every cereal crop, especially maize and wheat, as well as fruits and a variety of vegetables without any limitation (Majeed et al., 2015). It is located near 33.05° latitude and 74.82° longitude and falls under the sub-tropic zone. The entire valley of Samahni is approximately 55 km long and 10 km wide. It has north and south-facing high mountains, with 1000 km altitude and variable topography based on mountainous and semi-mountainous parts as well as agricultural fields (Wikipedia, 2023). The average maximum and minimum temperature are 28.9 °C and 15.8 °C respectively, and total average rainfall is approximately 1233 mm/year, with an average maximum and minimum humidity of 69.0% and 48.75%, respectively (Hussain, 2009). The soil of the experimental site was sandy loam and calcareous in nature, with comparatively low SOM and fertility status.

### 2.2 Isolation and characterization of plant growth promoting bacteria

To isolate PGPB, soil samples were collected in sterile plastic bags from three different sites of wheat and maize growing regions, Bhimber (BH), Samahni (SA), and Barnala (BA), and were stored at 4°C separately for further process (Dinesh et al., 2015). One gram of soil from each sample was dissolved into 10 mL of sterile water, shaken for 30 min at 150 rpm and 1 mL of supernatant was diluted 10-fold by serial dilution method. Next, 100 µL aliquots were spread on solid nutrient agar (NA) medium (composition is given in **Supplementary Table 1**) petri-plates which were already autoclaved at 121°C and 15 psi for 15 min. The petri-plates were incubated at 37°C for 3 days (Ogunmwonyi et al., 2008; Uthayasooriyan et al., 2016; Patel and Saraf, 2017; Albdaiwi et al., 2019; Manzoor et al., 2019; Li et al., 2021). The colonies from each plate were picked up and streaked on freshly prepared NA plates and the purified strains were stored on NA at 4°C (Kim et al., 2016).

### 2.3 Morphological and biochemical characterization of PGPB

The physical appearance of bacterial colonies was observed by the naked eye while morphological characterization was done through a light microscope and by adopting the Vincent method (Thirumal et al., 2017). These colonies were further characterized biochemically by adopting relative procedures. The oxidase activity of PGPB was determined by the oxidase test. A piece of filter paper was dipped in oxidase reagent while pure colonies were transferred to this filter paper with the help of a sterile needle. Catalase activity was determined by the CAT test by putting the pure culture of PGPB on a glass slide with one drop of 30% H_2_O_2_. Indole Acetic Acid (IAA) production and starch hydrolysis were determined through the procedure adopted by Hemraj et al., 2013 (Hemraj et al., 2013). For IAA, a culture of the PGPB colony was added into Tryptone Broth Medium (TBM) already autoclaved at 121°C and 15 psi for 15 min. The culture was incubated at 30 ± 2°C for 2 days. Furthermore, 1 mL of Kovac’s reagent was added and shaken gently every 10-15 min. Hydrolysis of starch by PGPB was observed by transferring a culture of colonies to the solid starch agar medium and incubating at 30 ± 2°C for 4 days, after which 5 mL iodine solution was added (Küpper et al., 2011; Hossain et al., 2019). Ammonia (NH_3_) production was assessed by taking culture in peptone broth, incubation at 30 ± 1°C for 48 hours, and the addition of 0.5 mL of Nessler’s reagent. Hydrogen cyanide (HCN) production was determined by placing culture on DF minimal salt media with the amendment of 4.4 g L^-1^ glycine. A piece of filter paper the size of the Petri plate was soaked in 0.5% picric acid and 2% sodium carbonate solution and placed in the Petri plate lid. After sealing the Petri plate with parafilm the media was incubated for 96 h at 28°C (Mumtaz et al., 2017). Siderophore production was determined by spotting the culture of selected colonies of PGPB on CAS medium without nutrients overlaying the top of the Nfb agar plates and incubating at 30°C for 48 hours. Phosphate solubilization was checked by bacterial strain spotted on SMRS1 medium with the composition of tricalcium phosphate, bromocresol, ammonium sulfate, and yeast extract and incubated for 7 days at 28°C (García et al., 2017).

GA production was tested by adding bacterial isolates in a nutrient broth medium and incubated at 37°C for seven days. After centrifugation at 8000 g for 10 min, a considerable portion of bacterial culture was added to 5 mL of zinc acetate. Furthermore, 2 mL of potassium ferrocyanide solution was added after 2 min and centrifuged again at 8000 g for 10 min. The supernatant was added to 30% HCl, incubated at 27°C for 75 min and absorbance was measured by spectrophotometer at 254 nm wavelength while GA production was calculated through standard curve (Kesaulya et al., 2015). Nitrate reduction was estimated by a small portion of culture that was added in a small quantity of semisolid medium (composition is given in **Supplementary Table 2**). The culture was incubated at 350 °C for 7 days and the reduction of nitrate to nitrite was tested by adding sulphanilic acid 8 g and naphthylamine 5 g each in 1000 mL of 5 N acetic acid (Narayan and Gupta, 2018).

### 2.4 Molecular characterization (16S rRNA gene analysis) of PGPB

The extraction of DNA from each isolate of all nine species was done with the help of the Power Soil^®^ DNA Isolation Kit (MO BIO, Carlsbad, CA, USA) (Azlan Halmi et al., 2018). The Genomic DNAs were sequenced at the National Institute of Health (NIH), Islamabad, Pakistan, where polymerase chain reaction (PCR) consisting of Taq DNA polymerase with appropriate forward and reverse primers was used to amplify 16S rRNA genes. The thermal cycling was based on the following steps: denaturing at 94°C for 5 minutes after which 30 cycles at 94°C for 1 min were conducted, and each reaction mixture was annealed and extended at 56°C and 72°C for 1 min, respectively. On the completion of these cycles, the reaction mixture was kept for 5 min at 72°C and then cooled down to 4°C. The automated sequencer (Genetic Analyzer 3130; Applied Biosystems, Foster City, CA, USA) was then used for sequencing. After obtaining proper nucleotide accession numbers, i.e., OP964678 (SA-7), OP976027 (BH-1), OP978159 (BA-3), OP978164 (BH-8), OP980571 (BA-6), OP978161 (SA-1), OP978163 (SA-5), OP978160 (BH-7), and OP978162 (BA-7), by submitting the related sequence to the gene bank of the National Center for Biotechnology Information (NCBI), the similarity of the resulting species with already identified bacterial species in genomic database was confirmed through blasting by utilizing the BLASTN tool of NCBI (Tamura et al., 2007; Kim et al., 2012; Gao et al., 2019). The latest version of MEGA 11 software was used to construct a neighbor-joining phylogenetic tree through the Jukes-Cantor method at 1000 bootstrap replications (Mukhuba et al., 2018; Tamura et al., 2021).

### 2.5 Inoculum preparation

After proper morphological, biochemical, and molecular characterization of PGPB, nutrient broth (NB) medium was prepared in three different 250 mL flasks, autoclaved at 121°C and 15 psi for 15 min. A combined culture from selected colonies of PGPB isolates was picked up from each plate separately with the help of a sterile loop and added into liquid NB medium incubated at 28 ± 2°C for 48-72 hours under shaking at 150 rev/min and maintained with 15% glycerol at -80°C (Kim et al., 2012; Pashapour et al., 2016; Trivedi et al., 2016). Furthermore, 95% ethanol was applied for 3 min to wheat seeds (*Triticum aestivum*, Chakwal-50) by shaking in a 10% solution of chlorox for 3 min and then washing with sterilized water. These sterilized seeds were soaked in PGPB inoculum for a few hours (Humaira and Asghari, 2013; Saadaoui et al., 2022).

### 2.6 Experimental design and treatments

A field was selected in Tehsil Samahni, District, Bhimber, AJK, and a composite soil sample was collected randomly from the whole field, followed by dividing the field into a total of 24 plots with 8 treatments and 3 replications, each 2m x 5m (10 m^2^) in size. The plot-to-plot and replication-to-replication distance was 1 m and 2 ft, respectively. Wheat (*Triticum aestivum*, Chakwal-50) seeds inoculated with native strains of PGPB (BA, BH, and SA) were sown by integrating DAP and FYM. The treatment combinations are shown in **Table 1**. The experiment was done under a complete randomized block design (RCBD) (Sedri et al., 2022).

**Table 1:** Treatment combinations of experiment.

Treatment	DAP kg h^-1^ + FYM t h^-1^	Seed inoculation with
**T_1_ (CK- Control)**	–	–
**T_2_**	150 + 10	–
**T_3_**	–	SA
**T_4_**	–	BH
**T_5_**	–	BA
**T_6_**	150 + 10	SA
**T_7_**	150 + 10	BH
**T_8_**	150 + 10	BA

### 2.7 Soil physicochemical analysis

The soil sample collected before sowing and after harvesting was analyzed for basic characteristics: textural class, soil moisture contents (SMC %), bulk density (BD g/cm^3^), soil organic matter (SOM %), EC_1:1_ (µS), CaCO_3_ (%), pH, total organic carbon (TOC %), total nitrogen (TN %), available phosphorus (ppm), and extractable potassium (ppm). The soil nutrients and their ratios were determined after soil sampling from each plot at three different depths, i.e., 0-15 cm, 15-30 cm, and 30-45 cm. For proper preparation, sampled soil was dried at room temperature and sieved through a 2 mm mesh (Asghar et al., 2013; Ansari and Deshmukh, 2017; Iram and Khan, 2018; Dai et al., 2021; Mukherjee et al., 2021). The relative percentage of sand, silt, and clay were analyzed through particle size distribution (ISO 11277, 2009) while soil textural class was confirmed by the texture triangle from the International Union of Soil Science (IUSS) (Moeys, 2018). SMC was checked through the oven dry method in which soil was placed in an oven at 105 °C for 48 hours (Saadaoui et al., 2022). BD was determined by the core sampler method (Iram and Khan, 2018). SOM and TOC were analyzed through the acid digestion method (Chen et al., 2019). Soil pH was estimated from a 1:1 soil suspension while for EC the extraction of this suspension was used. Both were tested through their respective glass electrodes and read out from pH and EC meters, respectively (Dandwate, 2020). CaCO_3_ was determined by the calcimeter method in which a soil sample was inoculated with 0.1 N HCl solution and volumes of CO_2_ were recorded then the percentage of CaCO_3_ was calculated (Elfaki et al., 2015). TN was investigated with the help of an elemental analyzer by dry combustion at 900 °C (Saadaoui et al., 2022). Available P was analyzed by Olsen’s method in which after soil extraction in a sodium bicarbonate solution, a mixture of ammonium molybdate, ascorbic acid, and a small amount of antimony tartrate was used for the development of color and absorbance was read at the 882 nm wavelength (Arshad et al., 2021). The AL method was used to analyze K which was determined by flame emission photometry (Latkovic et al., 2020).

### 2.8 Plant analysis

Plant growth and crop yield parameters such as plant height at maturity, root length (RL), shoot length (SL), root dry weight (RDW), shoot dry weight (SDW), root shoot ratio (R:S), spike length, no. of grains per spike, 1000-grain weight (GW), biological yield (BY), and economic yield (EY) (Akhtar et al., 2009) were recorded at the time of harvest while plant nutrients and biochemical parameters such as N, P, K, protein, carbohydrates, and oil content were determined by adopting proper procedures after the complete preparation of the plant sample. The grains from each treatment with three replications were selected and boiled with concentrated sulphuric and perchloric acids. The N content was determined by an elemental CNS analyzer (Vario model EL III), while P and K were analyzed using the wet combustion method and estimated with molybdate by a spectrophotometer and a flame emission photometer respectively. Protein was calculated by multiplying the amount of N by factor 5.83 which is for wheat only (Latkovic et al., 2020). The sample of wheat grains was ground into powder to determine carbohydrate and oil content. Carbohydrate was analyzed using the phenol and sulphuric acid method, observed through a spectrophotometer at 490 nm wavelength (Agrawal et al., 2011) while oil content was estimated by excessive extraction with petroleum ether and Soxhlet extractor (Kondić-Špika et al., 2019).

### 2.9 Statistical analysis

The data of all parameters was undergone through analysis of variance (ANOVA) while means were compared with the help of least significant difference (LSD) at 5% (p ≤ 0.05) probability level by Duncan’s test (Bargali et al., 1993). The results were correlated by Pearson’s correlation analyses (Mukherjee et al., 2021). The standard error mean of the data was represented in each figure by error bars (Akhtar et al., 2009).

## 3 Results and discussion

### 3.1 Basic physicochemical properties of soil

Soil physicochemical analysis demonstrated that the soils from the three different sites were sandy loam, loam, and clay loam, respectively, and were mostly calcareous with a pH range of 7.56-7.81. The major physical and chemical properties of these soils varied from each other (**Table 2**). The fluctuation in soil texture, pH, SOC, TN, AP, K, Ca, Mg, and Na, of the three different soils under different land use was quite clear (Oyedele et al., 2015). The soil of site 1, in Tehsil Samahni, exhibited the highest values of SMC, SOM, TOC, TN, AP, and K and is a mostly hilly area, with dense forest, low temperature, limited cultivation, and higher fertility. The soils from areas with low temperature, high moisture, and limited cultivations support the available SOM, TN, and TOC due to minimum loss (Wakene, 2001; Padalia et al., 2018). Site 2, in Tehsil Bhimber, was highest in BD, CaCO_3_, pH, and EC and is comparatively flat, with limited forests, high temperatures, excessive cultivation, and lower fertility. The parameters of site 3 were in between those of site 1 and site 2 (**Table 2**). These findings are in accordance with early investigations (Lemenih, 2004; Kizilkaya and Dengiz, 2010; Padalia et al., 2018), where the values of BD and pH were highest in soils of cultivated lands with excessive fertilizer use compared to uncultivated lands. The main factors involved in fluctuations of physiochemical properties from one area to another were differences in topography, climatic conditions, soil weathering, vegetation, microbial community (Paudel and Sah, 2003; Baumler, 2015; Bargali et al., 2018), and several biotic and abiotic processes (Bargali et al., 1992; Kondić-Špika et al., 2019).

**Table 2:** Soil physicochemical properties of experimental sites.

Properties	Values		
	Site 1	Site 2	Site 3
Textural Class	Sandy loam	Loam	Clay loam
Soil Moisture Content (%)	25	22	24
Bulk Density (g/cm^3^)	1.2	1.47	1.33
Organic Mater (%)	0.45	0.31	0.41
CaCO_3_ (%)	0.87	0.96	0.94
pH	7.56	7.81	7.72
EC_1;1_ (µS)	210	240	230
Total Organic Carbon (%)	10	6	5
Total Nitrogen (%)	1.1	0.87	0.94
Available Phosphorus (ppm)	11	8	9
Extractable Potassium (ppm)	123	115	110

### 3.2 Morphological and biochemical characteristics of PGPB

A total of 26 strains, 10, 8, and 8 from Samahni, Bhimber, and Barnala, respectively were extracted, out of which 9 strains, 3 from each site were selected for complete characterization. The morphological characterization of PGPB in our findings revealed that these strains were round, rod and spherical shaped, off-white, white-yellow, dark brown, pale pink, and blue-green in color, ranging from 0.75-7.5µm in size. All strains were motile and gram-negative although only SA-1 was gram-positive. The strain SA-1 was neutral, BH-7 and BA-6 were alkaline while all remaining strains were acidic in nature (**Table 3**). Based on morphological characterization, a total of 10 different PGPB isolates with diverse plant growth-promoting activities have been extracted from wheat fields (Sood et al., 2018). The native strains of PGPB have been characterized morphologically as they were mostly rod-shaped, gram-negative (Amezquita-Aviles et al., 2022), motile (Kalam et al., 2020), ranged from small to large size, and mostly acidic in nature with diverse colors (Santa Cruz-Suarez et al., 2021). The morphological variations and behaviors of native PGPB pointed out in the previous studies are very similar to our findings. Through biochemical characterization of PGPB strains, it was observed that oxidase was released by SA-1 and BA-7 while catalase was released by all strains except SA-5 and BA-7 (**Table 3**). The tests for oxidase and catalase were quite different among a large community of PGPB isolates as a considerable number of these isolates were positive and negative for these tests (Kesaulya et al., 2021). The strains SA-1, SA-5, BH-8, and BA-6 hydrolyzed starch as presented (**Table 3**). Biochemical analysis of PGPB strains showed that out of seven isolates, four hydrolyzed starch (Kalam et al., 2020). All selected strains were involved in IAA, GA, NH_3_, siderophores production, nitrate reduction, and phosphate solubilization (**Table 3**). Plant growth-promoting activities have been attributed to all ten strains which possessed the three main PGP traits: IAA, siderophore production, and P solubilization (Sheirdil et al., 2019). GA have been produced by all 37 isolates (Kesaulya et al., 2021). Approximately 200 strains were screened out, which were engaged in IAA, NH_3_ production, and P solubilization (Tsegaye et al., 2019). NH_3_ was produced by 79 out of 100 isolates (Dinesh et al., 2015) and all 15 isolates were involved in the production of NH_3_ (Thirumal et al., 2017). There were 20 different strains of PGPB involved in nitrate reduction (Narayan and Gupta, 2018). Similar evidence about nitrate reduction by PGPB isolates was obtained from the literature (Baliah and Rajalakshmi, 2015). In our findings, only the strain SA-1 was able to produce HCN which is very close to the observations of Tsegaye et al., 2019 (Tsegaye et al., 2019) where only 25 strains out of 200 produced HCN, and out of 36 isolates only 3 were positive for HCN production (Kesaulya et al., 2015). In contrast, many isolates produced it in a study conducted by Patel et al., 2017 (Patel and Saraf, 2017). The maximum IAA 54.3 µg/mL, GA1303 µg/mL, siderophores production 13.7 µg/mL and phosphate solubilization 47.6 µg/mL were related to strains BA-1,BH-7 and SA-5 while the minimum IAA 29.4 µg/mL, GA 490 µg/mL, siderophores production 3 µg/mL and phosphate solubilization 18.4 µg/mL were attributed by strains BH-8 and BH-1. The average IAA 45.3 µg/mL, GA 882 µg/mL, siderophores production 10.4 µg/mL and phosphate solubilization 31.9 µg/mL was done from SA, The location BH contributed the average IAA 41.7 µg/mL, GA 883 µg/mL, siderophores production 7.8 µg/mL and phosphate solubilization 32.7 µg/mL while location BA added the average IAA 36.13 µg/mL, GA 674 µg/mL, siderophores production 7.3 µg/mL and phosphate solubilization 30.3 µg/mL from SA, BH and BA, respectively (**Table 3**). The plant growth-promoting traits varied among PGPB strains with different ranges (Albdaiwi et al., 2019), for example, IAA ranges from 0.27–77.98 µg/mL (Majeed et al., 2015) and our results exist in this range which is very close to the findings of Thirumal et al., 2017 (Thirumal et al., 2017). Furthermore, GA has been found to range from 4322-1959 µg/mL (Gusmiaty et al., 2019) which is much higher than our observations but GA production by PGPB assessed by Afzal et al., 2008 (Afzal and Bano, 2008) is much closer to our results produced by all the isolates with varied concentrations (Kesaulya et al., 2021). Siderophores production and P solubilization range from 2.8-4.2 and 14.24-4.5 µg/mL, respectively (Kesaulya et al., 2015). The isolates SA-1, SA-5, and SA-7 were possibly matched with species *Bacillus*, *Stenotrophomonas*, and *Azosprillium*, respectively, and BH-1, BH-7, and BH-8 with *Azosprillium*, *Azotobacter*, and *Rhizobium*, respectively, while BA-3, BA-6, and BA-7 matched with *Azosprillium*, *Rhizobium*, and *Pseudomonas*, respectively (**Table 3**). The main bacterial species identified as PGPB strains were *Pseudomonas*, *Bacillus* (Kumar et al., 2012; Majeed et al., 2015; Sheirdil et al., 2019; Tsegaye et al., 2019), *Rhizobium* (Majeed et al., 2015; Santa Cruz-Suarez et al., 2021), *Azospirillum, Azotobacter*, and *Stenotrophomonas* (Majeed et al., 2015). The above-mentioned fluctuation in population, morphological, and biochemical characteristics, and plant growth-promoting activities of native strains of PGPB isolates as collected from three different tehsils of district Bhimber of AJK might be due to certain factors. The possible factors may be the stage of vegetation, site, soil sampling period, soil physical and chemical characteristics, environment (Chiarini et al., 1998; Donn et al., 2015), climate change, tillage practices, use of fertilizers (Mhete et al., 2020), and some other important abiotic stresses (Lopes et al., 2021).

**Table 3:** Morphological and biochemical characterization of native isolates.

Isolates	SA-1	SA-5	SA-7	BH-1	BH-7	BH-8	BA-3	BA-6	BA-7
Colony Color	Off-White	White-yellow	Off-White	Off-White	Dark Brown	Pale pink	Off-White	Pale pink	Blue-green
Size (µm)	0.75	5.0	3.0	3.0	5.5	2.0	3.0	2.0	7.5
Shape	Rod	Rod	Round	Round	Spherical	Rod	Round	Rod	Rod
Motility	+	+	+	+	+	+	+	+	+
Gram Staining	+	-	-	-	-	-	-	-	-
pH	Neutral	Acidic	Acidic	Acidic	Alkaline	Acidic	Acidic	Alkaline	Acidic
Oxidase	+	-	-	-	-	-	-	-	+
Catalase	+	-	+	+	+	+	+	+	-
Starch	+	+	-	-	-	+	-	+	-
IAA (µg/mL)	42.5	40.0	42.5	41.2	37.8	29.4	47.6	34.0	54.3
GA (µg/mL)	999.3	929.0	718.7	674.7	1303.0	490.0	741.0	519.0	788.3
NH_3_	+	+	+	+	+	+	+	+	+
HCN	+	-	-	-	-	-	-	-	-
Siderophores (µg/mL)	12.3	10.0	9.0	6.7	13.7	3.0	11.3	6.0	4.7
Nitrate reduction	+	+	+	+	+	+	+	+	+
Phosphate (µg/mL)	27.7	47.6	20.4	18.4	60.1	19.7	22.2	24.1	44.7
Suitable genus	*Bacillus*	*Strentrophomonas*	*Azosprillium*	*Azosprillium*	*Azotobacter*	*Rhizobium*	*Azosprillium*	*Rhizobium*	*Pseudomonas*

### 3.3 Molecular identification of PGPB based on 16S rRNA gene sequences

**Figure 1:**
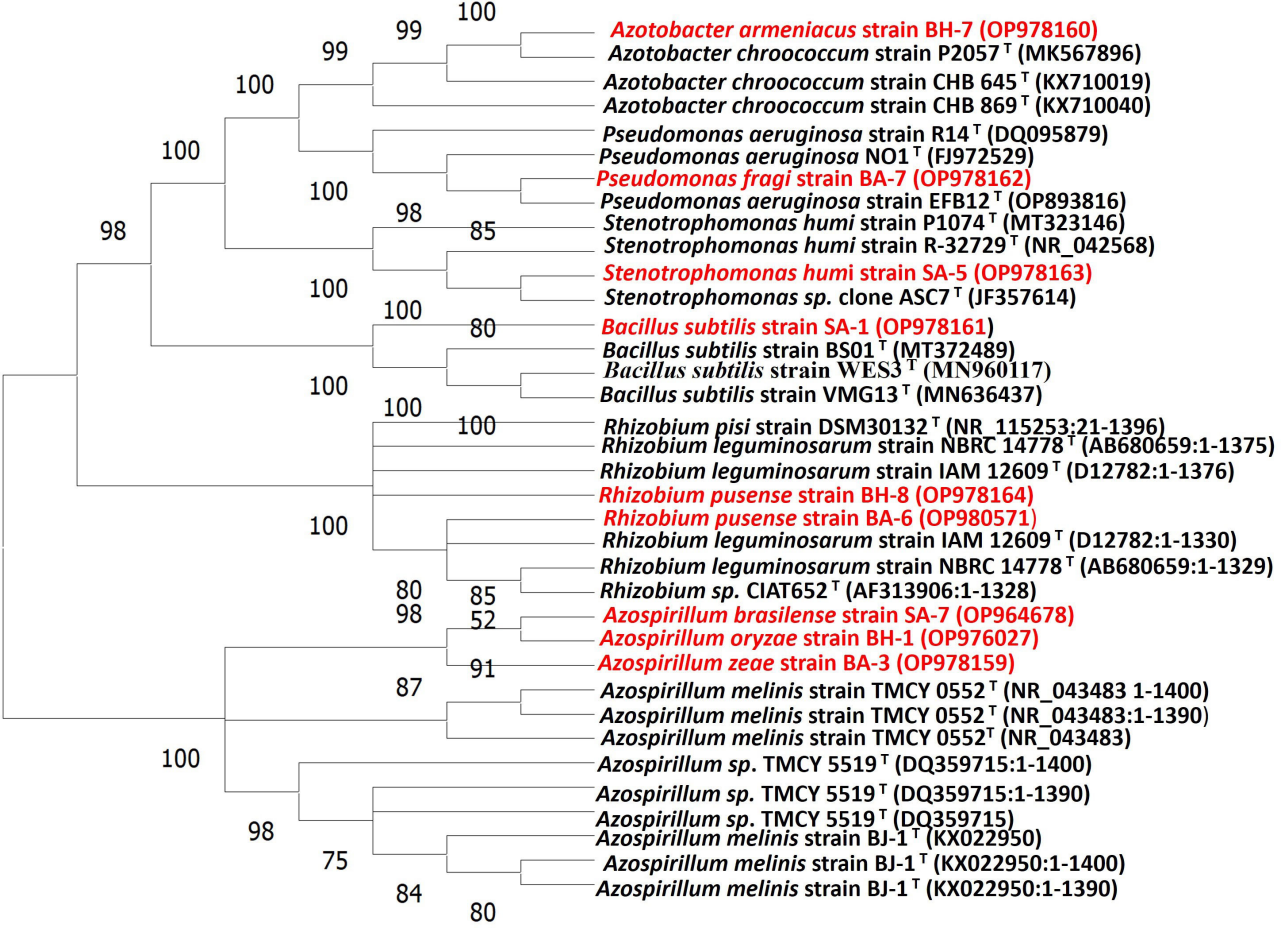
Phylogenetic tree of native isolates and their closest phylogenetic taxa inferred from neighbor-joining through 16S rRNA gene sequence analysis.

All nine strains were biochemically and morphologically characterized and further analyzed for molecular identification on 16S rRNA gene sequences level. The results revealed that three strains belonged to the genus *Azospirillum*: SA-7(*Azospirillum brasilense*), BH-1(*Azospirillum oryzae*), and BA-3 (*Azospirillum zeae*). Two strains were from the genus *Rhizobium* namely, BH-8, and BA-6 (*Rhizobium pusense*), and there was one strain from each genus *Bacillus, Stenotrophomonas, Azotobacter* and *Pseudomonas* which were SA-1 (*Bacillus subtilis*), SA-5 (*Stenotrophomonas humi*), BH-7 (*Azotobacter Armeniacus*), and BA-7 (*Pseudomonas fragi*) respectively (**Table 4**). The molecular identification based on 16S rRNA has been widely used to characterize a huge variety of native taxa of PGPB isolates from soils of various topography, soil and environmental conditions, and land use patterns (Majeed et al., 2015; Sheirdil et al., 2019; Kalam et al., 2020; Amezquita-Aviles et al., 2022). The nucleotide accession numbers were assigned to their respective PGPB strains. The assigning of an accession number to a particular identified soil bacterial species has been proven previously by Amezquita et al., 2022 and Kalam et al., 2020 (Kalam et al., 2020; Amezquita-Aviles et al., 2022). The phylogenetic tree was constructed on the basis of similarities between these identified species and closely related taxa obtained from a blast search (**Figure 1**). The 16S rRNA gene phylogenetic analysis represented species name, strain cod ID, nucleotide gene bank accession number, and relationship with closely related ancestor taxa (Kalam et al., 2020; Amezquita-Aviles et al., 2022). This similarity was more than 99% in all cases (**Table 4**). A similar trend has been observed by Majeed et al., 2015, Kalam et al., 2020, Sheirdil et al., 2019 (Majeed et al., 2015; Sheirdil et al., 2019; Kalam et al., 2020). The molecular identification and characterization of bacteria is a more effective way to differentiate closely related taxa which cannot be done by biochemical and morphological analysis. The 16S rRNA sequencing can determine every portion of an isolate at the molecular level. The authentication of 16S rRNA sequencing also has been proven by previous investigations which can identify species of taxa separately (Imran et al., 2010; Alam et al., 2011; Majeed et al., 2015).

**Table 4:** Molecular characterization of native isolates through 16S rRNA gene sequence analysis.

Selected Strains	Identified Taxa (Species)	Gene Size (bp)	Gene Bank Accession	Closely Related Taxa (Species)	Type Strains (Gene Bank ID)	Gene Bank Accession	Similarity (%)
SA-1	*Bacillus subtilis*	1519	OP978161	*Bacillus subtilis*	BS01^(T)^	MT372489	99.87
SA-5	*Stenotrophomonas humi*	1478	OP978163	*Stenotrophomonas humi*	R-32729^(T)^	NR_042568	99.86
SA-7	*Azospirillum brasilense*	1400	OP964678	*Azospirillum melinis*	TMCY 0552^(T)^	NR_043483	99.86
BH-1	*Azospirillum oryzae*	1390	OP976027	*Azospirillum* sp.	TMCY 5519^(T)^	DQ359715	99.21
BH-7	*Azotobacter armeniacus*	1392	OP978160	*Azotobacter chroococcum*	P205^(T)^	MK567896	99.86
BH-8	*Rhizobium pusense*	1376	OP978164	*Rhizobium leguminosarum*	IAM 12609^(T)^	D12782	99.85
BA-3	*Azospirillum zeae*	1372	OP978159	*Azospirillum melinis*	BJ-1^(T)^	KX022950	99.05
BA-6	*Rhizobium pusense*	1330	OP980571	*Rhizobium* sp.	CIAT652^(T)^	AF313906	99.40
BA-7	*Pseudomonas fragi*	1405	OP978162	*Pseudomonas aeruginosa*	NO1^(T)^	FJ972529	99.93

(All values are the average of three replicates).

### 3.4 Growth and yield of wheat

Regarding the difference in plant growth and crop yield parameters between CK and integrated dose, DAP+FYM+BA was significant but the overall difference among the means of all values was non-significant. Our results were consistent with the findings of Sheirdil et al., 2019 (Sheirdil et al., 2019) in which the plant growth and yield of wheat were significantly influenced by the combination of native strains of PGPB and chemical fertilizer as compared to the control in both pot and field trial experiments. The improvement in wheat growth and yield has been evidenced by other previous observations as wheat growth was significantly increased by bacterial isolates (Majeed et al., 2015) while the maximum yield was with the combination of chemical fertilizer and the most effective strains of PGPB as compared to the control. This efficacy might be due to a number of PGP traits of native strains which enhance root surface area, bunches of root hairs, and cortical cells which result in an improved ability of plants to take up more nutrients and hormones from soil (Sood et al., 2018). The highest values for plant height, no. of grains/spike, spike length, SL, RL, SDW, RDW, 1000 GW, BY, and EY were 90.7 cm, 87.7, 7.20 cm, 53.5 cm, 33.5 cm, 4.87 g, 1.32 g, 55.1 g, 8209 kg/h and 4572 kg/h, respectively, and were from the DAP+FYM+BA treatment while the lowest were 66.7 cm, 48.3, 4.13 cm, 21.9 cm, 10.3 cm, 1.53 g, 0.35 g, 23.1 g, 3498 kg/h and 2081 kg/h respectively, and were from CK. The values for R:S were 0.27 and 0.23 in the DAP+FYM+SA and CK treatments, respectively (**Table 5**). The plant growth of maize inoculated with *Bacillus safensis* PM22 was considerably improved as compared to non-inoculation (Amezquita-Aviles et al., 2022; Azeem et al., 2022). The overall ranking of these parameters under three main groups of treatment was CK< DAP+FYM< DAP+ FYM+ Different inoculations. A number of other studies have shown similar results (Adjanohoun et al., 2011; Jarak et al., 2012; Turan et al., 2012; Afzal et al., 2014). The results of our study showed that the combination of isolates BA-3, 6, 7 (*Azospirillum zeae*), *Rhizobium pusense* and *Pseudomonas fragi*), FYM and DAP was more effective for wheat growth and the individual effect of these isolates was also better as compared to others (Mihoub and Boukhalfa-Deraoui, 2014; Jamal et al., 2023). There were also some selective PGPB which have been screened out for their unique performance (Majeed et al., 2015; Amezquita-Aviles et al., 2022).

**Table 5:** Plant growth and crop yield parameters.

Parameters	Plant height (cm)	No. of grains/spike	Spike length (cm)	SL (cm)	RL (cm)	SDW (g)	RDW (g)	R: S	1000GW (g)	BY (kg)	EY (kg)
Treatments											
CK	66.7e	48.3e	4.13e	21.9f	10.3d	1.53e	0.35e	0.23a	23.1e	3498d	2081e
DAP+FYM	77.3bc	72.0bc	5.93cd	37.7bc	23.5bc	3.27bc	0.81bc	0.25a	42.4bc	6226b	3188bcd
SA	72.0cde	63.0cd	5.47d	29.6de	15.5cd	2.10d	0.61cd	0.29a	32.5d	5652bc	2568de
BH	69.7de	56.7de	4.73e	26.2ef	13.3d	1.7de	0.48de	0.29a	29.4d	4882c	2271e
BA	75.0cd	74.0b	5.8cd	32.3d	18.2cd	2.80c	0.74bc	0.27a	38.6c	5892bc	2855cde
DAP+FYM+SA	88.3a	80.7ab	6.47b	40.9b	29.8ab	3.63b	0.95b	0.26a	44.2b	6580b	3737ab
DAP+FYM+BH	81.7b	78.0b	6.20bc	35.0cd	27.9ab	3.23bc	0.93b	0.29a	41.0bc	6340b	3548bc
DAP+FYM+BA	90.7a	87.7a	7.20a	53.5a	33.5a	4.87a	1.32a	0.27a	55.1a	8209a	4572a

They are showing the differences among means.

### 3.5 Plant nutrients and quality parameters of wheat

**Figure 2:**
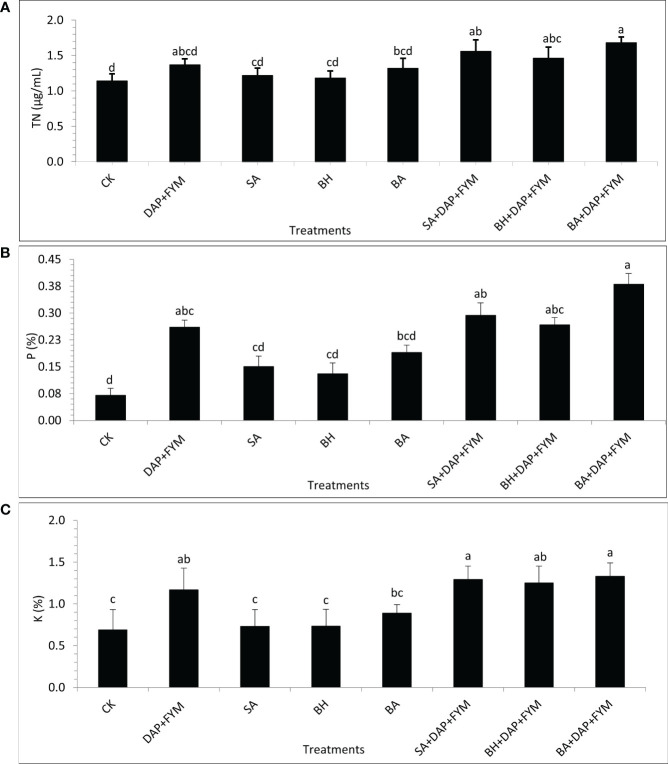
Plant nutrient concentration of wheat under different combinations of native strains of PGPB as biofertilizer; **(A)** Total Nitrogen (TN) [µg/mL]; **(B)** Phosphorus (P) [%]; **(C)** Potassium (K) [%]; means with similar letters are non-significantly different under Duncan’s test at *p* ≤ 0.05; factor: treatments; n= 3.

**Figure 3:**
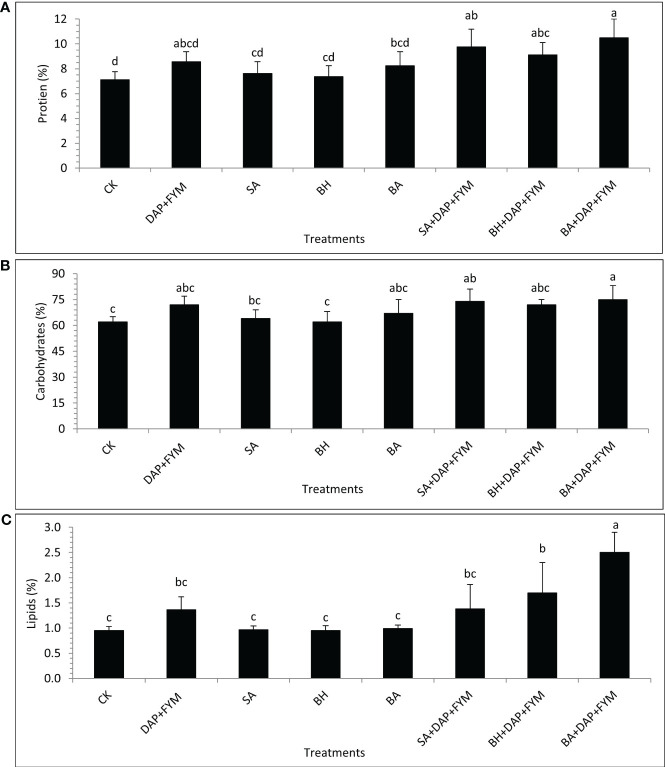
Plant quality parameters of wheat under different combinations of native strains of PGPB as biofertilizer; **(A)** Protein [%]; **(B)** Carbohydrates [%]; **(C)** Lipids [%]; means with similar letters are non-significantly different under Duncan’s test at *p* ≤ 0.05; factor: treatments; n= 3.

The overall difference between the means of plant nutrients was not significant but the effect of treatment with full dose DAP+ FYM+ inoculation was significantly different than CK. It was clear during the study that the improvement in nutrient NPK uptake by wheat plants when inoculated with PGPR and which were aided by efficient sources of nutrients such as fertilizers was quite significant compared to un-inoculated ones (Sood et al., 2018). The results revealed that the concentration of TN was highest in plants with treatment DAP+FYM+BA with a mean value of 1.68 µg/mL while it was found to be lowest in CK with a mean value of 1.14 µg/mL (**Figure 2A**). The percentage of plant P with the overall combination of DAP, FYM, and inoculation was significantly higher than without any treatment CK. However, this difference was non-significant in the case of all the inoculums applied separately without any combination with DAP and FYM. The mean percentage was highest, i.e., 3% in the case of DAP+FYM, DAP+FYM+SA, DAP+FYM+BH, and DAP+FYM+BA, lower, i.e., 0.16% in SA, BH, and BA alone and lowest i.e., 0.07% in CK (**Figure 2B**). K concentration showed the same trend as P. The difference of means under CK, SA, BH, and BA was not significant while that between CK and the treatments DAP+FYM, DAP+FYM+SA, DAP+FYM+BH, and DAP+FYM+BA was significant (**Figure 2C**). The improved plant nutrient content might be due to high soil nutrient concentration or non-associated fixation and solubilization resulting from plant growth-promoting processes (Sood et al., 2018) which can improve plant growth and crop yield (Asghar et al., 2002; Schoebitz et al., 2013; Kumar et al., 2014). The difference in means was not significant for the quality parameters of wheat crops. The effect of highly efficient inoculum with the combination of chemical and organic fertilizers such as DAP+FYM+BA on plant protein content was quite significant but case of the other six treatments, SA, BH, BA, DAP+FYM, DAP+FYM+SA, and DAP+FYM+BH, this difference was present but non-significant in comparison with CK (**Figure 3A**). The results of these observations were matched with the work of Parewa et al., 2021 (Parewa et al., 2021), as the quality parameters of wheat were highest under NPK, FYM, and bio-inoculant as compared to CK. The highest mean protein value of 10.5% was for DAP+FYM+BA and the lowest of 7.13% was for CK (**Figure 3A**). The maximum plant protein has been observed in plants inoculated by isolate PM22 as compared to un-inoculated with an up to 37% increase (Azeem et al., 2022). The nitrogen fixation and role of a number of bio-inoculants may increase protein content in wheat (Panwar and Ompal, 2000; Nabila et al., 2007). All three types of fertilizers, NPK, FYM, and bio-inoculant, have been involved in significant improvement of overall plant protein in wheat but the differences among all treatments were not significant (Parewa et al., 2021). Carbohydrate was maximum, i.e., 75%, in DAP+FYM+BA and minimum, i.e., 62% in CK. The difference between these two limits was significant although the difference between other treatments existed but was not significant (**Figure 3B**). The considerable increase in carbohydrates of wheat when treated with NPK, FYM, and bio-inoculants has been assessed when compared with no fertilizer such as CK (Parewa et al., 2021). In contrast to our findings, the differences among all treatments were significant. The overall percentage of lipids in wheat plants was lower compared to protein and carbohydrates. The average lipid of treatments was highest, 1.74%, in DAP+FYM, DAP+FYM+SA, DAP+FYM+BH, and DAP+FYM+BA, lower at 0.97% in SA, BH, and BA, and lowest, 0.95%, in CK. The difference in the means of lipids under CK and the combination of DAP, FYM, and inoculums of all native strains was significant but the difference between the strains SA, BH, and BA used separately, and CK and the other four treatments was non-significant (**Figure 3C**). The quality parameters of wheat have been significantly influenced by the combination of inorganic, organic, and bio-fertilizers (Parewa et al., 2021) which was also under different combinations and separate utilization of these three types of fertilizers (Saha et al., 2010; Kumar and Singh, 2013; Sharma et al., 2013; Parewa et al., 2021). The effect of native bio-inoculants in combination as well as separately was more prominent than different bio-inoculants; Consortia and VAM were involved in increasing protein in wheat (Parewa et al., 2021).

### 3.6 Soil nutrients

The effect of treatments DAP+FYM, SA, BH, and BA when used separately, on TOC and TN was significant while the effect of treatments with a combination of chemical, organic, and native strains of PGPB, DAP+FYM+SA, DAP+FYM+BH, and DAP+FYM+BA, was also clear but less than the effect previously described and was statistically non-significant (**Table 6**). The effect of treatments of different isolates on organic carbon was non-significant in wheat as shown by Sood et al., 2018 (Sood et al., 2018). The highest values of TOC and TN were obtained under the treatment of PGPB strain BH while the lowest was under CK. The effect of all treatments on available P was clear and quite significant but the difference of means among these treatments was not statistically significant. The maximum available P was provided by treatment DAP+FYM+BA while the minimum was obtained from CK (**Table 6**). Sood et al., 2018 (Sood et al., 2018) also screened out isolate B2 which is more prominent for improvement of available N and P in soil. They highlighted that, in field experiments, the joint application of PGPB inoculants of different isolates improved available N and P in soil significantly in which B2 isolate was also involved. The fixation of atmospheric N by symbiotic bacteria and mineralization of OM might be involved in the improvement of available N and P in soil. Ahmad et al., 2008 (Ahmad et al., 2008) also interpreted the same results. The difference in the means of C:N under all treatments was found to be non-significant. C:N was significantly increased in treatment using PGPB strain BH separately with the highest value of 7.28, while when combined with DAP+FYM, C:N was decreased to 4.09 at the lowest level. C:P was significantly increased by PGPB strains SA, BH, and BA when they were used separately, without DAP+FYM. The highest C:P was 20.7 in BA but was significantly lower, i.e., 11.9, in the combination treatment DAP+FYM+BA. The trend of N:P was reversed as it had a significant maximum value of 3.81 in CK and a minimum value of 3.04 in DAP+FYM+SA which was very close to the values from DAP+FYM+BH and DAP+FYM+BA, respectively (**Table 6**). The combined application of bacterial inoculant and chemical fertilizers visibly improved the soil nutrient concentration in wheat (Boukhalfa-Deraoui et al., 2015; Sood et al., 2018).

**Table 6:** Soil nutrient status under different treatments.

Parameters/ Treatments	Total Organic Carbon (%)	Total Nitrogen (%)	Available Phosphorus (ppm)	C: N	C: P	N: P
CK	4.89d	1.10b	2.99c	5.59bcd	18.0b	3.81a
DAP+FYM	6.69bc	1.34ab	4.3ab	5.94abc	16.0c	3.17bcd
SA	7.92ab	1.43a	4.39ab	6.33abc	19.1a	3.44bcd
BH	8.73a	1.47a	4.44ab	7.28a	20.2a	3.48abc
BA	7.40ab	1.36ab	3.74b	6.80ab	20.7a	3.56ab
SA+DAP+FYM	5. 94cd	1.37ab	4.33ab	5.22cd	14.2d	3.04cd
BH+DAP+FYM	5.32d	1.42a	4.48a	4.09d	12.1e	3.10cd
BA+DAP+FYM	5.51cd	1.44a	4.68a	4.37d	11.9e	3.01d

They are showing the differences among means.

**Figure 4:**
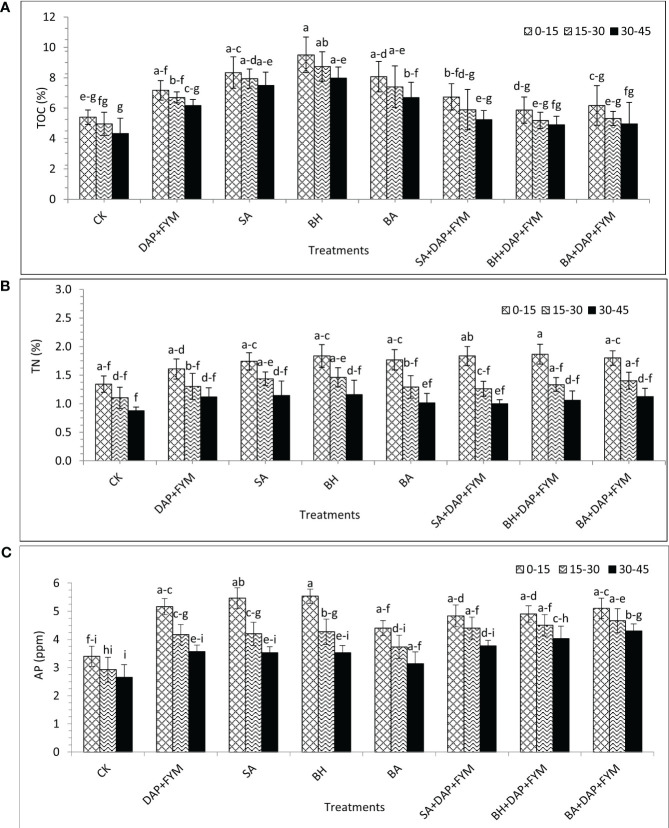
**(A)** Total organic carbon (TOC) at three different soil depths under eight different combinations of indigenous PGPB [%]; **(B)** Total nitrogen (TN) at three different soil depths under eight different combinations of indigenous PGPB [%]; **(C)** Available phosphorus(AP) at three different soil depths under eight different combinations of indigenous PGPB [ppm]; means with similar letters are non-significantly different under Duncan’s test at *p* ≤ 0.05; factors: treatments, depths; n= 3.

The difference in the means of TOC at three different depths was not significant, slightly significant in the case of C:N and N:P, and strongly significant for TN, available P, and N:P. The effect of depth on TOC, TN, and N:P was significantly increased from top to bottom with highest values of 7.16%, 1.72%, and 3.58, respectively, at the depth of 0-15cm as compared to the lowest values of 5.9%, 1.06%, and 3.06 at depth 30-45 cm, respectively. The trend of C:N and C:P was reversed as it increased from bottom to top with the highest values of 6.59 and 17.9 at the depth of 30-45 cm, respectively, and the lowest values of 4.76 and 15.2 at the depth of 0-15 cm, respectively. The effect of depth on available P was also highly significant. It was found to be the highest, i.e., 4.10 ppm at the depth of 15-30 cm and the lowest, i.e., 3.55 ppm at the depth of 30-45 cm (**Table 7**). The availability of basic nutrients was maximum at the surface of the soil because the 0-20 cm depth surface of the soil is richer in nutrients as compared to lower depths (Amgain et al., 2020). The interaction between treatments and depths for all soil nutrients was not significant and presented the same trend as described above in the case of treatments and depths individually (**Figures 4A–C**). The improvement in soil nutrient availability may be due to integrated nutrient management in which a number of microbes are involved in fixation, mineralization and solubilization etc (Sood et al., 2018). Our findings have been matched with a number of other studies (Aontoun et al., 1998; Riggs et al., 2001; Ogut and Er, 2016).

**Table 7:** Soil nutrients at different depths.

Parameters/Depths (cm)	Total Organic Carbon (%)	Total Nitrogen (%)	Available Phosphorus (ppm)	C: N	C: P	N: P
0-15	7.16a	1.72a	3.85a	4.76b	15.2c	3.58a
15-30	6.52ab	1.33b	4.10b	5.76a	16.4b	3.30b
30-45	5.97b	1.06c	3.55c	6.59a	17.9a	3.06b

They are showing the differences among means.

### 3.7 Correlation

The plant growth, crop yield, plant nutrients, and quality parameters were non-significantly (n=8, *p*>0.05) correlated with soil TOC. This correlation was positive only in case of R:S while negative for all other parameters. The correlation of R:S with soil TN was strongly significant (*p*<0.01) while R:S and BY were significantly (*p*<0.05) correlated with available P. All other parameters were non-significantly correlated with these two. This correlation was completely positive in all cases. There was a negative correlation of all parameters with soil C:N, C:P, and N: P, in which RL, EY, N, K, protein, carbohydrates, and lipids were significantly correlated with C:N while no. of grains/spike, R:S, and BY were non-significantly correlated with C:P but the remaining were significantly and strongly significantly correlated. R:S was non-significantly and lipids were significantly correlated while the remaining were highly significantly correlated with N:P (**Table 8**). The maximum plant parameters were non-significantly correlated with most soil nutrients (Azlan Halmi et al., 2018). Similar observations were made by Li et al., 2016 and Yusuf et al., 2009 (Yusuf et al., 2009; Li et al., 2016). But this relationship was quietly significant in the findings of Sood et al., 2018 (Sood et al., 2018).

**Table 8:** Correlations (Pearson) between soil nutrients, plant growth, crop yield, and growth-promoting parameters.

Soil nutrients	TOC	TN	AP	C: N	C: P	N: P
Plant parameters						
Plant height	-0.42	0.38	0.61	-0.69	-0.82*	-0.90**
No. of grains/spike	-0.27	0.48	0.64	-0.58	-0.70	-0.88**
Spike length	-0.26	0.51	0.69	-0.60	-0.72*	-0.91**
SL	-0.32	0.41	0.64	-0.60	-0.74*	-0.85**
RL	-0.44	0.39	0.64	-0.74*	-0.87**	-0.94**
SDW	-0.42	0.34	0.57	-0.66	-0.78*	-0.85**
RDW	-0.37	0.45	0.65	-0.68	-0.79*	-0.88**
R: S	0.56	0.90**	0.71*	0.10	0.02	-0.33*
1000GW	-0.28	0.46	0.66	-0.57	-0.72*	-0.87**
BY	-0.15	0.60	0.77*	-0.52	-0.67	-0.89**
EY	-0.44	0.38	0.62	-0.73*	-0.84**	-0.89**
N	-0.46	0.35	0.58	-0.72*	-0.83**	-0.88**
P	-0.33	0.45	0.69	-0.64	-0.79*	-0.93**
K	-0.51	0.27	0.56	-0.75*	-0.88**	-0.90**
Protein	-0.46	0.35	0.58	-0.72*	-0.83**	-0.88**
Carbohydrates	-0.48	0.26	0.55	-0.71*	-0.84**	-0.90**
Lipid	-0.52	0.31	0.55	-0.79*	-0.87**	-0.75*

**Correlation is Strongly significant while *significant at 0.05 level.

## 4 Conclusion

The extensive application of inorganic fertilizer alone is dangerous for the environment as compared to integration with bio- and organic fertilizers. This study has demonstrated the morphological, biochemical, and molecular characterizations of native strains of PGPB in detail. The reduction of toxic effects of chemical fertilizers by the addition of indigenous PGPB isolates in integrated form could provide a sustainable alternative and an effective solution to control the degradation of the environment and help promote sustainable agriculture. The results showed that the combination of more efficient native PGPB strains with organic and inorganic compounds is suitable for crop sustainability and soil nutrient retention which may indirectly rehabilitate adverse soil conditions and a polluted environment. PGPB strains containing bio-fertilizers are beneficial as they increase nutrient uptake in plants by increasing the mobility of nutrients in the rhizosphere region. The results of the study also showed that the combined application of these bacterial strains and fertilizers increased the nutrient concentration in wheat crops. Another reason for using these bio-fertilizers is their cost-effectiveness and eco-friendliness as compared to chemical fertilizers. Studies have shown that the cost of these bio-fertilizers is comparatively three times less than chemical fertilizers, making them suitable for farmers to utilize for their crops (Kumar et al., 2022). Bio-fertilizers are effective as they provide profit to farmers by increasing the crop yield and reducing the cost of chemical fertilizers spent on the crops (Naveed et al., 2015). However, one of the limitations of these biofertilizers is their short shelf life. It can be prolonged by converting them into liquid fertilizers, by lyophilization or cryopreservation of strains, and by using strains that are genetically modified to be drought and thermo-tolerant (Mahmud et al., 2021). The shelf life of PGPB strains depends on various factors and typically ranges between 6 months to 2 years or more as per the preservation method used and the type of strain (Bashan et al., 2013). Research is further required to overcome the limitations of these bio-fertilizers so that their potential can be utilized at maximum.

## Data availability statement

The datasets presented in this study can be found in online repositories. The names of the repository/repositories and accession number(s) can be found in the article/**Supplementary Material**.

## Author contributions

Conceptualization: IA, MAh, MF, and MI; Experimentation: IA and MAh; Data Analysis and Review: IA, MAh, MAk, AU, AA, and MJ; Original Draft preparation: IA and MAh; Review, Editing and Manuscript Finalization: MF, MAr, and AN. All authors contributed to the article and approved the submitted version.
