# Supplementary material for: Characterizing indigenous plant growth promoting bacteria and their synergistic effects with organic and chemical fertilizers on wheat (Triticum aestivum)
Source: Front Plant Sci. 2023 Aug 16;14:1232271. doi: 10.3389/fpls.2023.1232271 (PMC10505817; doi:10.3389/fpls.2023.1232271)
Supplement: Supplementary file 1 [file Table_1.docx]

Supplementary Material

**Characterizing Indigenous Plant Growth-Promoting Bacteria and their Synergistic Effects with Organic and Chemical Fertilizers on Wheat (*Triticum aestivum*)**

# Supplementary Tables

**Supplementary Table 1. Composition of Nutrient Agar Medium**

| Component | Quantity per Liter of water |
| --- | --- |
| Peptone | 5 g |
| NaCl | 5 g |
| Beef extract | 1.5 g |
| Yeast extract | 1.5 g |
| Agar | 15 g |

**Supplementary Table 2. Composition of Semi-solid Medium for Nitrate Reduction**

| **Component** | **Quantity** |
| --- | --- |
| Malic acid | 5 g |
| KOH | 4 g |
| K_2_HPO_4_ | 0.50 g |
| MgSO_4_.7H_2_O | 0.20 g |
| NaCl | 0.10 g |
| CaCl_2_ | 0.02 g |
| FeSO_4_.7H_2_O | 0.50 g |
| MnSO_4_. H_2_O | 0.10 g |
| Na_2_MoO_4_.2H_2_O | 0.002 g |
| Bromothymol blue | (0.5% in 95% alcohol) 2.0 ml |
| Agar | 1.75 g |
| NH_4_NO_3_ | 10 mM |
| Distilled water | 1 liter |
